# Single-cell longitudinal analysis of SARS-CoV-2 infection in human airway epithelium identifies target cells, alterations in gene expression, and cell state changes

**DOI:** 10.1371/journal.pbio.3001143

**Published:** 2021-03-17

**Authors:** Neal G. Ravindra, Mia Madel Alfajaro, Victor Gasque, Nicholas C. Huston, Han Wan, Klara Szigeti-Buck, Yuki Yasumoto, Allison M. Greaney, Victoria Habet, Ryan D. Chow, Jennifer S. Chen, Jin Wei, Renata B. Filler, Bao Wang, Guilin Wang, Laura E. Niklason, Ruth R. Montgomery, Stephanie C. Eisenbarth, Sidi Chen, Adam Williams, Akiko Iwasaki, Tamas L. Horvath, Ellen F. Foxman, Richard W. Pierce, Anna Marie Pyle, David van Dijk, Craig B. Wilen

**Affiliations:** 1 Cardiovascular Research Center, Section of Cardiovascular Medicine, Department of Internal Medicine, Yale School Medicine, New Haven, Connecticut, United States of America; 2 Department of Computer Science, Yale University, New Haven, Connecticut, United States of America; 3 Department of Laboratory Medicine, Yale University, New Haven, Connecticut, United States of America; 4 Department of Immunobiology, Yale University, New Haven, Connecticut, United States of America; 5 Universite Claude Bernard Lyon 1, Faculte de Medecine Lyon Est, Lyon, France; 6 Department de Bioinformatique, Univ Evry, Universite Paris-Saclay, Paris, France; 7 Department of Molecular Biophysics & Biochemistry, Yale School of Medicine, New Haven, Connecticut, United States of America; 8 Department of Molecular, Cellular, and Developmental Biology, Yale School of Medicine, New Haven, Connecticut, United States of America; 9 Department of Comparative Medicine, Yale School of Medicine, New Haven, Connecticut, United States of America; 10 Department of Neuroscience, Yale School of Medicine, New Haven, Connecticut, United States of America; 11 Department of Obstetrics, Gynecology, and Reproductive Sciences, Yale School of Medicine, New Haven, Connecticut, United of States of America; 12 Department of Biomedical Engineering, Yale University, New Haven, Connecticut, United States of America; 13 Department of Pediatrics, Yale School of Medicine, New Haven, Connecticut, United States of America; 14 Department of Genetics, Yale School of Medicine, New Haven, Connecticut, United States of America; 15 Yale Center for Genome Analysis, Yale School of Medicine, New Haven, Connecticut, United States of America; 16 Department of Anesthesiology, Yale University, New Haven, Connecticut, United States of America; 17 Department of Internal Medicine, Yale School of Medicine, New Haven, Connecticut, United States of America; 18 The Jackson Laboratory, Farmington, Connecticut, United States of America; 19 Howard Hughes Medical Institute, Chevy Chase, Maryland, United States of America; New York University School of Medicine, UNITED STATES

## Abstract

There are currently limited Food and Drug Administration (FDA)-approved drugs and vaccines for the treatment or prevention of Coronavirus Disease 2019 (COVID-19). Enhanced understanding of Severe Acute Respiratory Syndrome Coronavirus 2 (SARS-CoV-2) infection and pathogenesis is critical for the development of therapeutics. To provide insight into viral replication, cell tropism, and host–viral interactions of SARS-CoV-2, we performed single-cell (sc) RNA sequencing (RNA-seq) of experimentally infected human bronchial epithelial cells (HBECs) in air–liquid interface (ALI) cultures over a time course. This revealed novel polyadenylated viral transcripts and highlighted ciliated cells as a major target at the onset of infection, which we confirmed by electron and immunofluorescence microscopy. Over the course of infection, the cell tropism of SARS-CoV-2 expands to other epithelial cell types including basal and club cells. Infection induces cell-intrinsic expression of type I and type III interferons (IFNs) and interleukin (IL)-6 but not IL-1. This results in expression of interferon-stimulated genes (ISGs) in both infected and bystander cells. This provides a detailed characterization of genes, cell types, and cell state changes associated with SARS-CoV-2 infection in the human airway.

## Abstract 2

Single-cell analysis of human airway epithelial cells infected with SARS-CoV-2 provides novel insights into viral replication, cell tropism, and host-viral interactions. This study reveals novel polyadenylated viral transcripts, preferential tropism for ciliated cells that later extends to other epithelial cell types, and cell-intrinsic expression of type I and type III IFNs and IL6 induced by infection, resulting in expression of interferon-stimulated genes in both infected and bystander cells.

## Introduction

In December 2019, a novel viral pneumonia, now referred to as Coronavirus Disease 2019 (COVID-19), was observed in Wuhan, China [1]. Severe Acute Respiratory Syndrome (SARS) Coronavirus (CoV) 2, the causative agent of COVID-19, has caused a global pandemic. There are currently no approved drugs or vaccines for the treatment or prevention of COVID-19. Enhanced understanding of viral pathogenesis at the cellular and molecular level is critical for the development of prognostic tools and novel therapeutics. CoVs are enveloped viruses with positive-sense, single-stranded RNA genomes ranging from 26 to 30 kb [2]. Six human CoVs have been previously identified: HCoV-NL63 and HCoV-229E, which belong to the Alphacoronavirus genus; and HCoV-OC43, HCoV-HKU1, SARS-CoV-1, and Middle East Respiratory Syndrome CoV (MERS-CoV), which belong to the Betacoronavirus genus [3]. In the past 2 decades, CoVs have become a major public health concern due to potential zoonotic transmission, as revealed by the emergence of SARS-CoV in 2002, which infected approximately 8,000 people worldwide with a mortality rate of approximately 10%, and MERS-CoV between 2012 and 2020, which infected 2,500 people with a mortality rate of approximately 36%, and now SARS-CoV-2, with an estimated mortality rate of approximately 1% [4,5]. SARS-CoV-2 infection is characterized by a variable presentation with common symptoms including fever, cough, and malaise [6,7]. Severe cases of COVID-19 progress to acute respiratory distress and acute lung injury that can lead to death [8,9].

Tissue and cell tropism are key determinants of viral pathogenesis. SARS-CoV-2 entry into cells depends on the binding of the viral spike (S) protein to its cognate receptor angiotensin converting enzyme II (ACE2) on the cell surface [6,10]. ACE2 is also the receptor for SARS-CoV-1 and HCoV-NL63, yet these viruses induce distinct morbidity and mortality, suggesting unknown determinants of coronavirus pathogenesis [11,12]. Additionally, proteolytic priming of the S protein by host proteases is also critical for viral entry [10]. The cellular protease transmembrane protease serine 2 (TMPRSS2) cleaves and primes the SARS-CoV-2 S protein [10,13,14]. Endosomal cysteine proteases cathepsin B and cathepsin L are also sufficient to prime the S protein [15,16]. Another host protease, furin, has been shown to cleave the S protein at the S1/S2 site to mediate SARS-CoV-2 entry into cells [17–19]; however, the precise role of host proteases in SARS-CoV-2 entry remains to be determined [20,21].

COVID-19 patients have increased levels of pro-inflammatory effector cytokines, such as tumor necrosis factor alpha (TNF*α*), interleukin (IL)-1B, and IL-6, as well as chemokines, such as CCL2 and CXCL10, especially in those who are critically ill [22–25]. These studies suggest that an overexuberant immune response characterized by cytokine storm rather than direct virus-induced damage may be responsible for COVID-19 pathogenesis. The cell types and mechanisms underlying this immune response are unclear for SARS-CoV-2. In addition, it has been observed that age is a strong risk factor for more severe disease [26]. In the United States of America, between February 12 and March 16, 2020, the case fatality rate was 10.4% to 27.3% for patients 85 years old, compared to 0.1% to 0.2% for patients 20 to 44 years old [26]. The reasons for this increased risk remain unknown.

Our knowledge of SARS-CoV-2 biology and pathogenesis is incomplete. To address this gap, we performed single-cell (sc) RNA sequencing (RNA-seq) on organotypic human bronchial epithelial cells (HBECs) infected with SARS-CoV-2. This culture system supports epithelial cell differentiation and mimics key aspects of the mucosal epithelium. By utilizing scRNA-seq, electron microscopy, and immunofluorescence microscopy, we revealed that ciliated cells are a major target of SARS-CoV-2 infection in primary bronchial epithelial cells. During the course of infection, cell tropism of SARS-CoV-2 extended to other epithelial cells including basal and club cells. Furthermore, SARS-CoV-2 infection elicited cell-intrinsic expression of type I interferons (IFNs), type III IFNs, and IL-6 but not IL-1. Interferon-stimulated gene (ISG) expression was observed in both infected and bystander cell populations. Here, we provide a detailed analysis of SARS-CoV-2 infection in HBECs which reveals novel SARS-CoV-2 transcripts, identifies preferential tropism for ciliated cells, and characterizes host gene expression and cell states related to infection.

## Results

### Dynamics of SARS-CoV-2 infection in primary human bronchial epithelial cells

**Fig 1:**
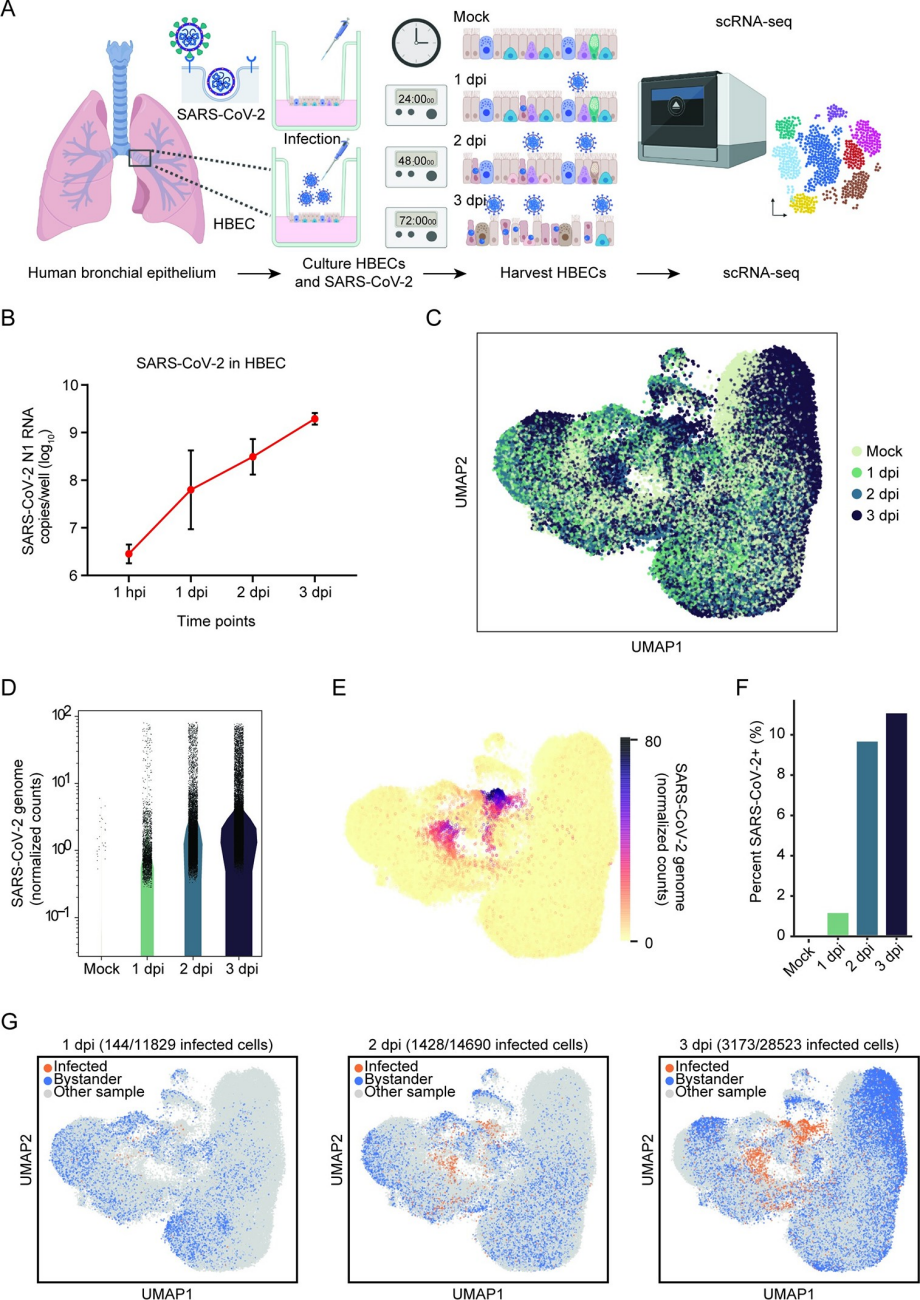
scRNA-seq reveals SARS-CoV-2 infection in HBECs. **(A)** Experimental process for longitudinal scRNA-seq. HBECs in ALI cultures were mock-infected or infected with 10^4^ PFU of SARS-CoV-2. Cells were then harvested at 1, 2, and 3 dpi and processed via scRNA-seq using the 10x Genomics platform. **(B)** HBEC ALI cultures were infected with 10^4^ PFU SARS-CoV-2 and harvested at 1 hpi, 1, 2, and 3 dpi. Viral transcripts were detected by real-time quantitative PCR using primers specific for the nucleocapsid gene. **(C)** UMAP visualization of cells after batch correction with BB-kNN. Each dot represents a cell; color represents the given time post-infection. **(D)** Normalized and square-root transformed counts of the SARS-CoV-2 viral genome in mock, 1, 2, and 3 dpi samples. Viral counts in each cell were determined by aligning reads to a single, genome-wide reference. **(E)** UMAP visualization of the normalized and square-root transformed counts of SARS-CoV-2 reads (color). **(F)** Percent of cells infected by SARS-CoV-2 in mock, 1, 2, and 3 dpi; cells were considered infected if they had greater than 10 SARS-CoV-2 full-length genome counts. **(G)** UMAP visualizations of infected (orange) and bystander cells (blue) in each time point after batch correction. The number of infected cells over time is indicated in each time point. Bystander cells are defined as cells that remain uninfected in HBEC samples challenged with SARS-CoV-2. The individual numerical value per condition for C–G is listed in S1 Data. Illustration for Fig 1A was created using BioRender.com. ALI, air–liquid interface; BB-kNN, batch-balanced kNN; dpi, days post-infection; HBEC, human bronchial epithelial cell; hpi, hour post-infection; PFU, plaque forming unit; SARS-CoV-2, Severe Acute Respiratory Syndrome Coronavirus 2; scRNA-seq, single-cell RNA sequencing; UMAP, Uniform Manifold Approximation and Projection.

To characterize SARS-CoV-2 interaction with the human airway, we performed scRNA-seq of SARS-CoV-2–infected airway epithelium. We cultured primary HBECs at an air–liquid interface (ALI) for 28 days and then challenged the apical surface of the epithelium with 10^4^ plaque forming units (PFUs) of SARS-CoV-2 (USA/WA1-2020) (Fig 1A). Exponential viral replication over the course of the experiment was demonstrated by quantitative real-time polymerase chain reaction (qRT-PCR) of cell lysate for the SARS-CoV-2 nucleocapsid (N) gene (Fig 1B). At 1, 2, and 3 days post-infection (dpi), a single-cell suspension was generated, and 3′ scRNA-seq was performed on 77,143 cells across 4 samples with an average of 31,383 reads per cell (Fig 1C, S1 Data). To define SARS-CoV-2–infected cells, we mapped reads to the viral reference genome and quantified viral transcript abundance on a per cell basis across the conditions (Fig 1D). We defined productively infected cells as those with at least 10 viral transcripts per cell, which controls for background due to misaligned reads in the mock sample. Mapping viral counts per cell showed an increase in SARS-CoV-2 transcripts during the course of infection (Fig 1D) as represented by the dense colored clusters in the Uniform Manifold Approximation and Projection (UMAP; Fig 1E). Consistent with viral genome replication (Fig 1B), we observed a time-dependent increase in the abundance of infected cells from 1 to 3 dpi (Fig 1F). Mapping of productive infected cells reveals multiple infected cell clusters that expand over time and are not present in the mock sample (Fig 1G). These results further validate that primary HBECs are permissible to SARS-CoV-2 and can be used as a platform to recapitulate the conditions found in the human airway.

### SARS-CoV-2 transcriptome in primary human bronchial epithelial cells reveal unique noncanonical reads

**Fig 2:**
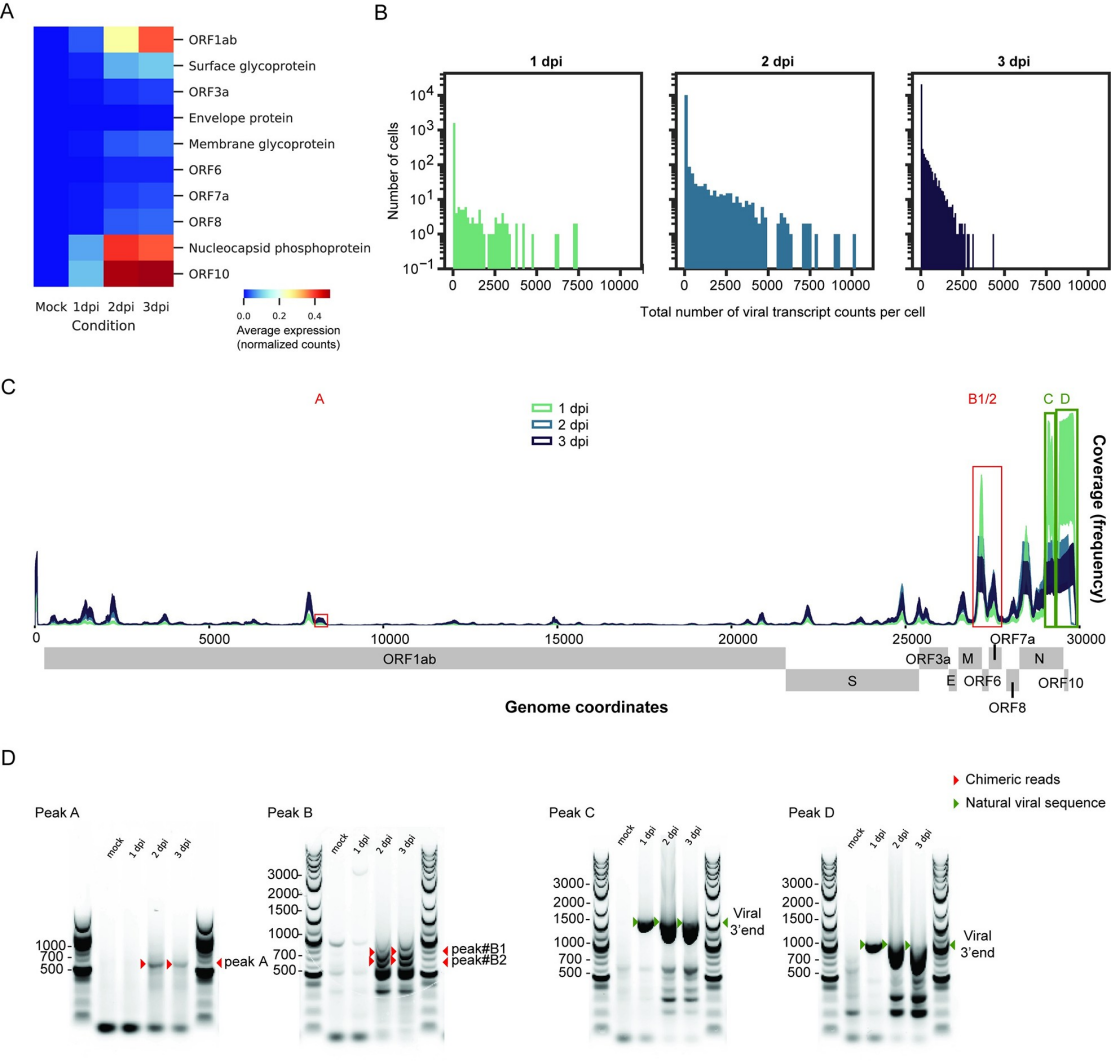
SARS-CoV-2 transcriptome analysis reveals noncanonical transcripts. **(A)** Heatmap showing average expression (normalized and square-root transformed counts) for reads aligned to individual viral ORFs at each time point. **(B)** Histograms of viral transcript raw counts per cell on a logarithmic scale for 1, 2, and 3 dpi. **(C)** Coverage plot of SARS-CoV-2 transcriptome at the scRNA-seq level. The sequencing depth was computed for each genomic position for each time point. The coverage showed both canonical (near the 3′ end, green boxes) and noncanonical (near the 5′ end) poly-adenylated sgRNAs. Two unique peaks were identified (red boxes). The individual numerical value per condition for A–C is listed in S1 Data. **(D)** RT-PCR spanning the junctions between poly-A tails and SARS-CoV-2 genome body for noncanonical transcripts (Peaks A, B1/2, red boxes) and 2 positive controls (Peaks C, D, green boxes). The products were run on agarose gels. Red arrowheads denote the expected amplicons for novel transcripts, while green arrowheads denote amplicons for the natural viral 3′ end. The raw images for D can be found in S1 Raw Images. dpi, days post-infection; ORF, open reading frame; RT-PCR, reverse transcription PCR; SARS-CoV-2, Severe Acute Respiratory Syndrome Coronavirus 2; scRNA-seq, single-cell RNA sequencing; sgRNA, subgenomic RNA.

Next, we characterized the SARS-CoV-2 transcriptome at the single-cell level across the different conditions in HBECs (Fig 2, S1 Data). Increased detection of SARS-CoV-2 open reading frame (ORF) counts were observed in a time-dependent manner; in particular, ORF1ab, nucleocapsid, and ORF10 were highly detected (Fig 2A). We then investigated the distribution of total viral transcript counts per cell over the course of infection. In parallel with the detection of individual ORFs (Fig 2A), increases in total viral transcript counts per cell were observed throughout the course of infection (Fig 2B), consistent with active viral replication. In addition, the distribution of polyadenylated viral transcripts shifts from 3′ to 5′ during the infection time course (Fig 2C). Furthermore, aside from the viral reads expected to align immediately upstream of the canonical SARS-CoV-2 poly-A tail, our results show additional reads aligning elsewhere in the viral genome suggesting the existence of noncanonical, poly-adenylated subgenomic RNAs (sgRNAs) (Fig 2C). The distribution of polyadenylated viral transcripts shifts from 3′ to 5′ during the course of infection (Fig 2C). Using reverse transcription PCR (RT-PCR), we successfully validate 2 unique peaks (red open boxes), one peak (A) that mapped in the middle of the ORF1ab region and a second peak (B1/2) that mapped near the ORF6 boundary (Fig 2C). Our results confirm that RT-PCR products corresponding to each of the 2 peaks appear after 2 dpi (Fig 2D, red arrows). Importantly, the absence of these RT-PCR bands in the mock and 1 dpi samples suggests that they are not the result of nonspecific oligo-d(T) priming of cellular or viral RNAs. We included 2 positive controls (green open boxes), one peak (C) that mapped near the middle region of nucleoprotein (N) and another peak (D) at the 3′ end and amplifying RT-PCR products of increasing length from the canonical SARS-CoV-2 poly-A tail (Fig 2D, bottom panel, green arrows). These RT-PCR bands that appear as early as 1 dpi are specific to infected cells and run at their expected lengths. These positive controls validate that we are able to capture known poly-adenylated viral transcripts with this RT-PCR priming strategy. This is consistent with a recent SARS-CoV-2 transcriptome study [27].

### SARS-CoV-2 preferentially infects ciliated cells in primary human bronchial epithelial cells

**Fig 3:**
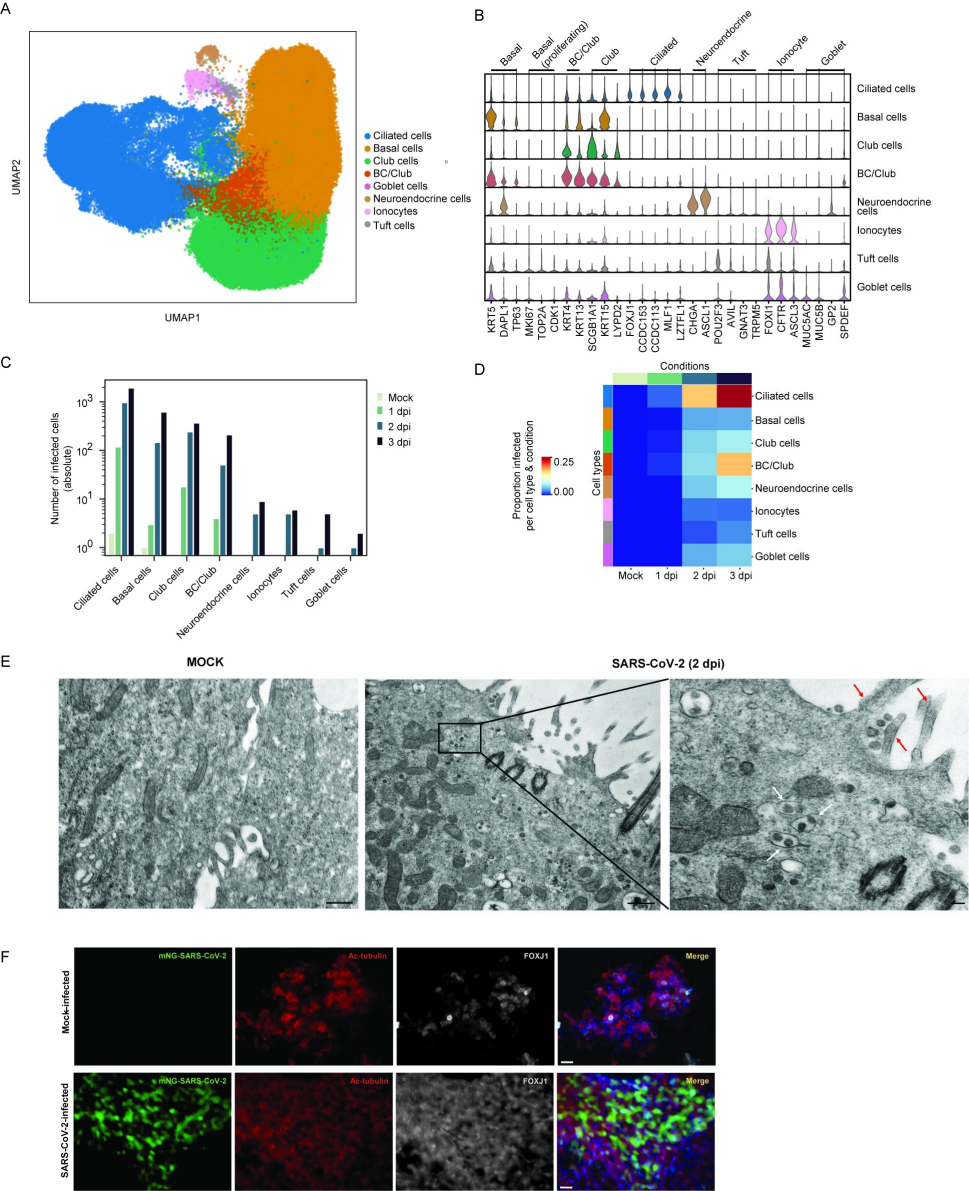
SARS-CoV-2 tropism in primary HBECs reveals ciliated cells as primary target cells. **(A)** UMAP visualization of the cell clusters after manual annotation. The UMAP projections of the dataset are color coded by cell type. Eight distinct cell clusters were identified: ciliated, basal, club, a mixture of basal and club cells (BC/Club), goblet, neuroendocrine, ionocytes, and tuft cells. **(B)** Louvain clusters were annotated with a cell type based on enrichment of canonical cell type markers (see Materials and methods). Violin plots show range-scaled expression (normalized and square-root transformed counts) of marker genes across clusters. **(C)** Reads mapping to the full SARS-CoV-2 genome were mapped to each of the 8 distinct cell types; cells with greater than or equal to 10 viral transcript counts were considered infected. The absolute number of infected cells in each cell type is plotted and stratified by time point (color). **(D)** Heatmap, where each row and column represents the proportion of infected cells in a particular cell type and condition (color per row and column represents the number of infected cells divided by the total number of cells in that particular subset). Conditions are color coded as indicated in Fig 1C, and cell types are color coded as depicted in Fig 3A. The individual numerical value per condition for A–D is listed in S1 Data. **(E)** Transmission electron microscopy image of mock (left) and SARS-CoV-2 HBEC reveal infected ciliated cells at 2 dpi (right). Scale bars correspond to 500 nm. White arrows denote virus particles, and red arrows denote cilia. **(F)** Immunofluorescence assay of mock- and mNeon-Green SARS-CoV-2 on differentiated HBECs stained with Ac-tubulin (red) and FOXJ1 (white), known markers for ciliated cells. Scale bars correspond to 25 μm. The raw images for E can be found in S2 Raw Images, and raw images for F can be found in S3 Raw Images. Ac-tubulin, acetylated tubulin; dpi, days post-infection; FOXJ1, Forkhead Box J1; HBEC, human bronchial epithelial cell; SARS-CoV-2, Severe Acute Respiratory Syndrome Coronavirus 2; UMAP, Uniform Manifold Approximation and Projection.

The human airway is composed of diverse epithelial cell types with critical functions in gas exchange, structure, and immunity. We sought to determine the cell tropism of SARS-CoV-2 in the bronchial epithelium, as the airway is a critical target of viral pathogenesis. We identified 8 major clusters comprising canonical epithelial cell types: ciliated cells, basal cells, club cells, goblet cells, neuroendocrine cells, ionocytes, and tuft cells (Fig 3A, S1 Data). We also observed a cell population intermediate between basal cells and club cells (BC/club) likely representing basal stem cells differentiating into club cells. Analysis of differentially expressed genes (DEGs) shows that these cell clusters express classical epithelial cell type–specific markers (Fig 3B). Mapping viral infected cells within specified epithelial cell types reveals that ciliated, basal, club, and BC/Club cells are susceptible to SARS-CoV-2 infection, whereas goblet, neuroendocrine, tuft cells, and ionocytes are relatively resistant to infection (Fig 3C and 3D). At 1 dpi, ciliated cells represent approximately 84% of infected cells and continue to comprise the majority of infected cells throughout infection (Fig 3C, S1A–S1D Fig). However, during productive infection, the number of infected basal, club, and BC/club cells also increase, suggesting that these cells are significant secondary target cells (Fig 3C, S1C and S1D Fig). The distribution of polyadenylated viral transcripts along the length of the genome is similar across infected cell types in which nucleocapsid phosphoprotein and ORF10 were the top viral genes identified (S1E Fig). To independently verify SARS-CoV-2 cell tropism, HBECs cultured under identical conditions as for scRNA-seq were assessed by transmission electron microscopy. At 2 dpi, we observed numerous virus particles approximately 80 nm in size in ciliated cells (Fig 3E, right panel). This is consistent with the size and morphology of coronaviruses [28]. These particles were not observed in a mock control sample (Fig 3E, left panel). To further validate ciliated cell tropism by SARS-CoV-2, we infected HBECs with a SARS-CoV-2 mNeonGreen reporter virus and 2 dpi co-stained for the ciliated cell markers FOXJ1 and acetylated tubulin (Ac-tubulin) [29–31]. SARS-CoV-2 positive cells colocalized with FOXJ1 positive cells (Fig 3F). Together, this confirms that ciliated cells are a major target of SARS-CoV-2 in the initial phase of infection but not the sole cell type infected in the human bronchus.

### Determinants of cell tropism of SARS-CoV-2 in primary human bronchial epithelial cells

**Fig 4:**
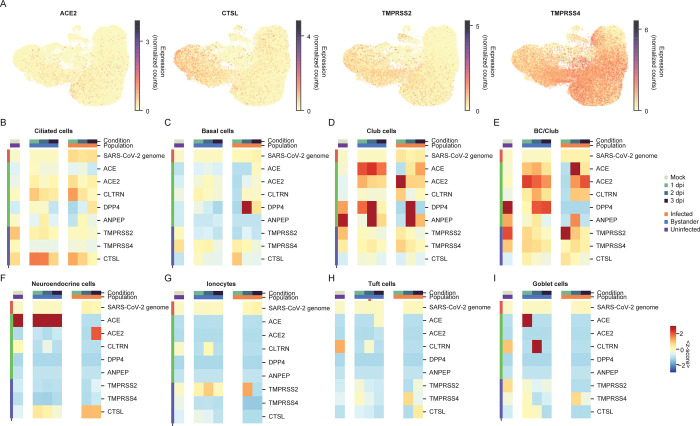
Expression of known entry determinants across bronchial epithelial cell types during SARS-CoV-2 infection. **(A)** UMAP visualization of HBEC samples, colored by expression (normalized and square-root transformed counts) of the ACE2 receptor, CTSL, TMPRSS2, and TMPRSS4 proteases. **(B–I)** Heatmaps comparing average expression (represented as a z-score, where each cells’ expression is transformed by subtracting the average and dividing by the standard deviation across the entire dataset) of genes homologous to ACE2 (ACE, ANPEP, and CLTRN) or relevant to other coronaviruses (DPP4; MERS-CoV receptor and ANPEP; and 229E receptor), in ciliated (B), basal (C), club (D), BC/Club cells (E), neuroendocrine (F), ionocytes (G), tuft cells (H), and goblet cells (I) in infected, bystander, and uninfected cells at different time points. The average is calculated with respect to cells in infected, bystander, and uninfected cells in mock, 1, 2, and 3 dpi (color bar legend atop heatmaps). The color scale shows the average expression (represented as z-score) for each cell type and condition. The individual numerical value per condition for A–I is listed in S1 Data. ACE2, angiotensin converting enzyme II; dpi, days post-infection; HBEC, human bronchial epithelial cell; SARS-CoV-2, Severe Acute Respiratory Syndrome Coronavirus 2; TMPRSS2, transmembrane protease serine 2; UMAP, Uniform Manifold Approximation and Projection.

Next, we sought to determine the host transcriptional correlates of SARS-CoV-2 cell tropism. As viral entry is a major determinant of cell tropism, we first investigated whether expression of ACE2, the SARS-CoV-2 receptor, predicted infection. We observed ACE2 expression at low levels across ciliated, basal, club and BC/club cells in the mock condition (Fig 4A, S1 Data). ACE2 expression is a significant predictor of infection at the cell type level as ACE2 expression was increased in the 4 susceptible cell populations: ciliated (Fig 4B), basal (Fig 4C), club (Fig 4D), and BC/club (Fig 4E) relative to the non-susceptible cell types: neuroendocrine (Fig 4F), ionocytes (Fig 4G), tuft cells (Fig 4H), and goblet cells (Fig 4I). Yet, ACE2 expression was poorly correlated with SARS-CoV-2 infection on a per cell basis (Spearman’s r between viral genome and ACE2 in ciliated cells, rho = −0.06). ACE2 was recently demonstrated to be an ISG [32]. However, we do not observe a significant increase in ACE2 expression in either infected or bystander cells relative to the mock sample (Fig 4E–4H).

To examine whether expression of other potentially proviral genes explain SARS-CoV-2 cell tropism, we assessed the expression of the proteases that may potentiate SARS-CoV-2 infection. The transmembrane serine protease TMPRSS2 and cathepsin L have been implicated in SARS-CoV-2 entry [10]. We also examined the related protease TMPRSS4, which cleaves influenza hemagglutinin, similar to TMPRSS2, and may also play a role in SARS-CoV-2 entry [33,34]. TMPRSS2 and CTSL were expressed predominantly in basal, club, and ciliated cells, while TMPRSS4 was broadly expressed in all epithelial cell types (Fig 4A–4E). The specific role of proteases in governing SARS-CoV-2 tropism in the human airway epithelium remains to be further elucidated.

### Innate immune response to SARS-CoV-2 infection

**Fig 5:**
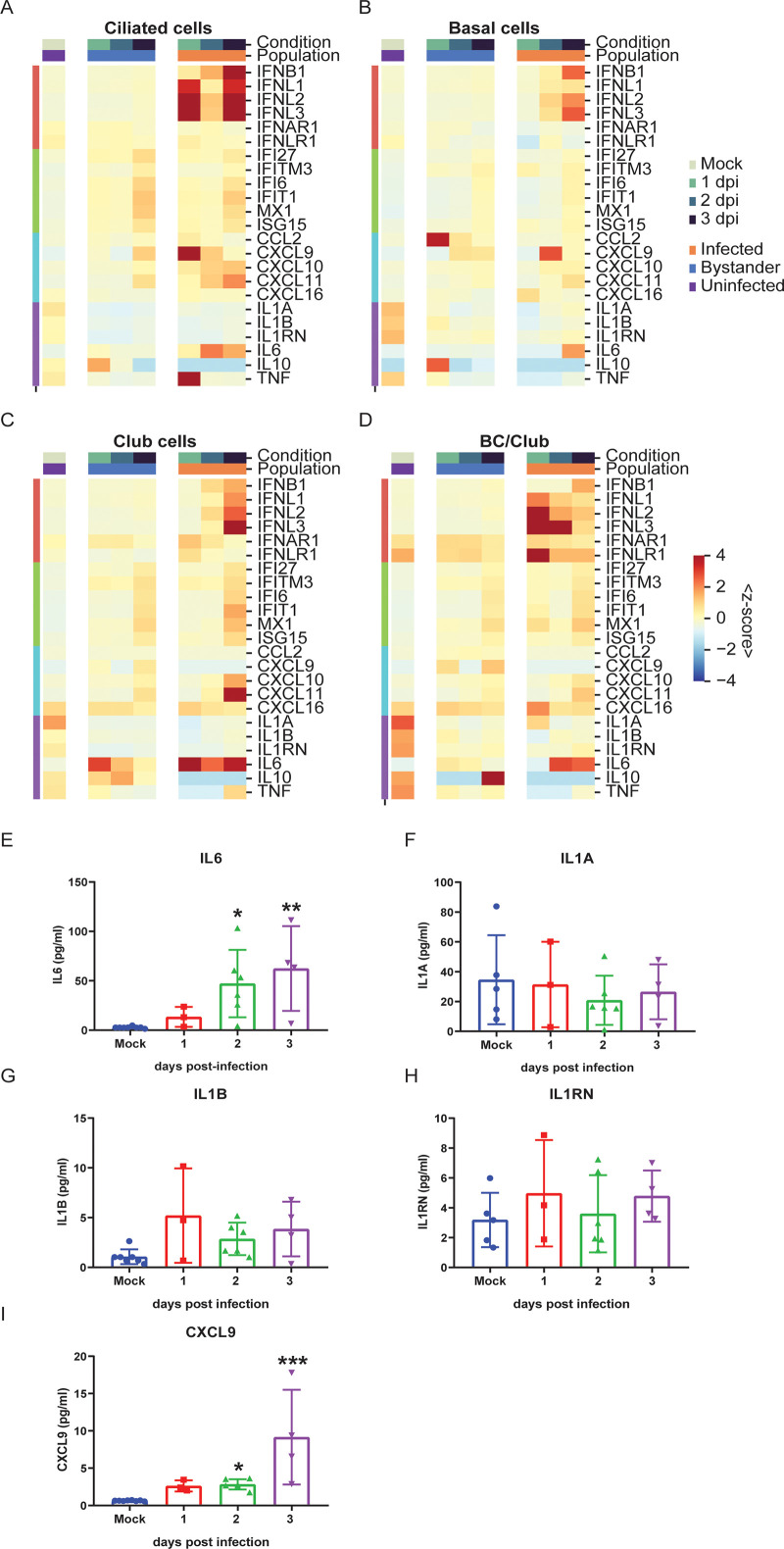
SARS-CoV-2 infection induces a robust innate immune response. **(A–D)** Heatmaps showing average expression (represented as z-score) of key innate immune and inflammatory genes in ciliated (A), basal (B), club (C), and BC/club cells (D) in infected, bystander, and uninfected cells in different time points (color bar legend atop heatmaps). Rows index average expression for type I and III IFNs and chemokines (left color bar legend). The individual numerical value per condition for A–D is listed in S1 Data. **(E–I)** Cytokine and chemokine measurement in basolateral supernatants of HBEC cultures infected with SARS-CoV-2 at 0 (mock), 1, 2, and 3 dpi from 3 independent experiments. SARS-CoV-2 infection induces IL-6 (E) and CXCL9 (I) secretions. Minimal changes in IL-1A (F), IL-1B (G), and IL-1RN (H) secretions are observed. All statistical analysis was performed using Prism GraphPad version 8. Significance compared to mock infection was analyzed using nonparametric Kruskal–Wallis test, indicated with a bar, and the *p*-value is represented by a symbol (*p < 0.05, **p < 0.01, ***p < 0.001). dpi, days post-infection; HBEC, human bronchial epithelial cell; IFN, interferon; IL, interleukin; SARS-CoV-2, Severe Acute Respiratory Syndrome Coronavirus 2; TNF, tumor necrosis factor.

We investigated the host transcriptome to assess the host immune response to SARS-CoV-2 infection at single-cell resolution in the human airway epithelium. We observed robust induction of both type I IFN (IFNB1) and type III IFNs (IFNL1, IFNL2, and IFNL3) in ciliated, basal, club, and BC/club (Figs 5A–4D, S1 Data) cells co-expressing SARS-CoV-2 transcripts. Interestingly, the kinetics of IFNB1 induction were delayed relative to type III IFN. In contrast, there was scant IFN induction in uninfected ciliated, basal, club, and BC/club cells (Figs 5A–4D). This demonstrates that direct SARS-CoV-2 infection of a given cell is critical for IFN induction. Type I and III IFNs signal through IFNAR and IFNLR, respectively, resulting in the expression of hundreds of ISGs. Consistent with this, we observed broad ISG induction (IFI27, IFITM3, IFI6, MX1, and ISG15) in both infected and bystander cells of all cell types (Figs 5A–4D, S3A–S3D Fig), suggesting that IFN from infected cells is acting in trans on both infected cells and uninfected bystander cells.

The host antiviral response also results in chemokine induction, leading to recruitment of immune cells, a hallmark of severe COVID-19 [35]. Here, we observe induction of CXCL9, CXCL10, and CXCL11 which propagate signals through the cognate CXCR3 receptor to recruit activated T cells and NK cells (Fig 5A–5D). This induction was evident in infected but not in bystander cells (Fig 5). In contrast, CCL2 and CXCL16 which recruit monocytes and T cells, respectively, were not dynamically regulated over the conditions evaluated (Fig 5A–5D). We also observed substantial induction of the pro-inflammatory cytokine IL-6 in infected ciliated, basal, club, and BC/club cells but not in uninfected bystander cells of these same populations (Fig 5A–5D). Interestingly, expression of pro-inflammatory IL-1 was modestly down-regulated in all cell types after infection, whereas IL-10 and TNF*α* expressions were not significantly regulated by infection in this system (Fig 5A–5D). We further characterized the levels of secreted cytokines and chemokines in the basolateral supernatant of mock and infected HBECs and observed induction of IL-6 and CXCL9 but not IL-1A, IL-1B, and IL-1RN, consistent with gene expression changes (Fig 5E–5I).

### Differentially expressed genes in response to SARS-CoV-2 infection

**Fig 6:**
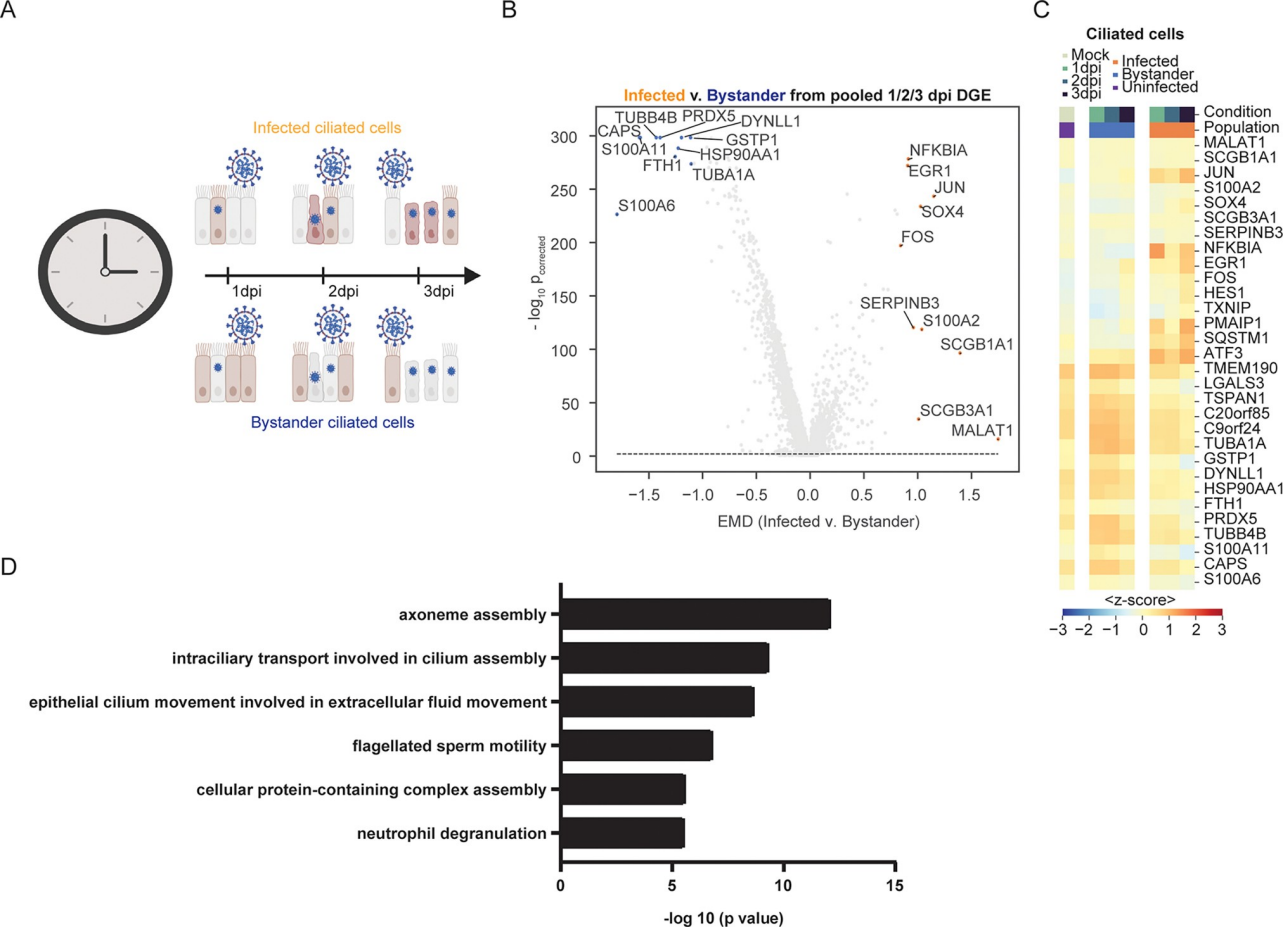
Expression of DEGs in ciliated cells in response to SARS-CoV-2 infection. **(A)** Schematic of the differential expression analysis comparing ciliated cells from the infected and bystander populations. **(B)** Volcano plots highlighting the most DEGs between infected and bystander populations in ciliated cells pooled from 1, 2, and 3 dpi samples, as ranked by the EMD. The y-axis shows the negative log base-10, Benjamini–Hochberg corrected *p*-value from a Mann–Whitney U test with a continuity correction, comparing the expression between infected and bystander. The dashed line shows the significance, set at p_corrected_ ≤ 0.01 (see Materials and methods). **(C)** Heatmap showing the average expression (represented as z-score) in each condition (color bar legend atop heatmap) of the top 15 differentially up-regulated and top 15 down-regulated genes from the analysis in Fig 6A and 6B. **(D)** Pathway analysis of top and bottom 200 DEGs which are significantly down-regulated in SARS-CoV-2–infected versus bystander ciliated cells. The analysis was done using the PANTHER GO tool with significance assessed using Fisher exact test. The individual numerical value per condition for A–D is listed in S1 Data. The raw data for generating B–D are listed in S3 Data. Illustration for Fig 6A was created using BioRender.com. DEG, differentially expressed gene; dpi, days post-infection; EMD, Earth Mover’s Distance; SARS-CoV-2, Severe Acute Respiratory Syndrome Coronavirus 2.

To determine how SARS-CoV-2 infection perturbs the cellular transcriptome, we computationally pooled the 3 infected samples and analyzed the top 100 DEGs between infected and uninfected bystander cells of a given cell type within the 1, 2, and 3 dpi samples (Fig 6A). We also analyzed the top 30 DEGs per time point on the 4 major cell types (ciliated, basal, club, and BC/club) comparing the infected and bystander cells (S3A Fig). PANTHER gene ontology analysis revealed that infected ciliated cells versus bystander cells had significant down-regulation of genes included in biological processes involved in cilium function (e.g., DYNLL1), calcium signaling (e.g., CALM1 and CALM2), and iron homeostasis (e.g., FTH1 and FTL; Fig 6B and 6C, S3A Fig). Increased expression of genes involved in apoptosis (e.g., PMAIP1, SQSTM1, and ATF3), translation initiation and viral gene expression (e.g., RPS12 and RPL37A), and inflammation (e.g., NFKBIA and NFKBIZ) were observed in infected ciliated cells compared to bystander cells (Fig 6B and 6C, S3A–S3C Fig). We observed similar results when time points were analyzed in isolation (S4A–S4C Fig, S2 Data). We then analyzed the top and bottom 200 genes of SARS-CoV-2–infected versus bystander ciliated cells (S3 Data) to explore up-regulation and down-regulation of cellular pathways. This highlighted significant down-regulation of cilium assembly and motility pathways during SARS-CoV-2 infection (Fig 6D).

## Discussion

To effectively treat COVID-19, we must first understand how SARS-CoV-2 causes disease and why the clinical presentation varies from asymptomatic infection to lethal disease. Here, we report a longitudinal single-cell transcriptomic analysis of SARS-CoV-2 in infected organotypic HBECs that recapitulates the orientation and complexity of the airway epithelium. Primary human airway epithelial cells have the ability to differentiate into multiple cell types that cannot be attained using 2D cultures, thus making this system a more physiologic relevant model to study host–pathogen interactions as well as mechanisms involved in respiratory diseases. Our study shows that in primary HBEC cultures, ciliated cells are the major target cell of SARS-CoV-2 infection at the onset of infection and that cell tropism expands to basal, club, and BC/club over time. Identification of the cell types infected by SARS-CoV-2 informs pathogenesis. Ciliated cells, which are abundant in the respiratory epithelium, are involved in the mucociliary clearance in the human lung that helps propel harmful aerosols, pathogens, and toxins in the lower respiratory tract [36]. Our finding that ciliated cells are the predominant target cell of SARS-CoV-2 at the onset of infection in primary bronchial epithelium has several important implications. First, dysfunction of ciliated cells by infection by SARS-CoV-2 may impair mucociliary clearance and increase the likelihood of secondary infection. Second, asthma, chronic obstructive pulmonary disease, and smoking are associated with both cilia dysfunction and increased severity of COVID-19 [37]. Whether these underlying conditions alter ciliated cells and thus increase their susceptibility to infection remains unclear. SARS-CoV-2–infected ciliated cells have been identified in autopsied and hospitalized patients [38,39], highlighting a potential connection between ciliated cells susceptibility to SARS-CoV-2 infection and COVID-19 disease progression. An important determinant of SARS-CoV-2 infection is receptor recognition. ACE2 is used by SARS-CoV-2 as a receptor for entry and the proteases TMPRSS2 and cathepsin L for priming the S protein [10]. ACE2 expression was relatively low in the present dataset, consistent with independent studies [40–42]. Levels of ACE2 poorly correlate with SARS-CoV-2 susceptibility on a per cell basis. This is consistent with low levels of ACE2 being sufficient for infection by SARS-CoV-2 and the limitation in detecting low abundance transcripts by single-cell methods. Subcellular localization of ACE2 to the motile cilia on the apical surface of airway epithelial cells may facilitate infection of cells with low levels of ACE2 expression [43]. ACE2 expression levels and dynamics may play an important role in COVID-19 pathogenesis in vivo, although not observed here. Specifically, given ACE2 is an ISG, it is intriguing to speculate that SARS-CoV-2 infection induces IFN, which, in turn, induces ACE2 expression, creating a positive feedback cycle to amplify infection and promote disease [44,45]. ACE2 can also be regulated by noninfectious factors which may contribute to COVID-19 pathogenesis. In particular, cigarette smoke caused a dose-dependent up-regulation of ACE2 expression, potentially explaining the susceptibility of smokers to SARS-CoV-2 infection [37].

Our data reveal several novel viral transcripts, and our methodology differentiated infected from bystander cells. Single-cell transcriptomics enabled us to elucidate the SARS-CoV-2 transcriptome at single-cell resolution in multiple primary cell types over time. We also identified polyadenylated viral transcripts remote from the 3′ end of the viral genome, which was unexpected given our sequencing method. Our RT-PCR validation experiments confirm the production of at least 2 unique transcription regulatory sequence (TR)S-independent transcripts with poly-A tails that do not appear to result from nonspecific oligo-d(T) priming. As the reported recombination rate for coronaviruses is high [46,47], it is possible that these short reads correspond to nonspecific polymerase jumping. However, recent studies have identified TRS-independent chimeric RNAs produced during SARS-CoV-2 infection of Vero cells, a small portion (1.5%) of which are fused in frame [27]. Taken together with our results, this may suggest that noncanonical sgRNAs with coding potential are produced during SARS-CoV-2 infection; however, this would require further validation. HCoV-229E nonstructural protein 8 (nsp8) was recently shown to possess template-independent adenyltransferase activity [48]. Because poly-A tails play important roles in the stability and translation potential of canonical SARS-CoV-2 sgRNAs, it is interesting to speculate that coronaviruses might rely on the production of noncanonical, poly-adenylated sgRNAs to serve as decoys for cellular deadenylases. This would result in preservation of the poly-A tails of the genomic and sgRNAs. Indeed, the production of sgRNAs during flaviviral infections is important for resistance to cellular exoribonucleases and innate immune evasion [49,50].

COVID-19 pathogenesis is characterized by a lag following viral transmission with symptom onset at day 7 and disease severity peaking 14 dpi [51,52]. This is in contrast to seasonal human coronaviruses and implicates an important role for the host immune response in COVID-19 progression. Several recent studies have revealed that induction of innate immunity during SARS-CoV-2 infection is dependent on viral replication kinetics and multiplicity of infection (MOI) [53,54]. IFN responses that promote ISGs are important defense mechanisms. Here, we show that the innate response to SARS-CoV-2 is intact and rapid, as characterized by induction of IFN in infected cells resulted in broad ISG expression in both infected and bystander cells. We also observed potent induction of the pro-inflammatory cytokine IL-6 and chemokines, which likely contribute to the inflammatory response in vivo [53]. Consistent with this, IL-6 is a potent pro-inflammatory cytokine, and serum IL-6 levels predict respiratory failure [55]. IL-6 was also found to be elevated in response to SARS-CoV-2 in cell culture, animal models, and bronchoalveolar lavage fluid of COVID-19 patient samples [24,56]. In contrast, we did not see any significant change in the levels of IL-1RN or IL-1RA in the infected HBECs which was observed in other cell and animal models [56]. The discrepancy may be due to species or cell type differences. Therapies targeting the IL-6 receptor are currently in clinical trials for the treatment of COVID-19 [57].

By assessing the transcriptome of SARS-CoV-2–infected bronchial epithelial cells, we observed up-regulation of genes involved in inflammation, apoptosis, and translation initiation and viral gene expression. In contrast, we detected down-regulation of genes involved in cilium function, calcium signaling, and iron homeostasis. Interestingly, while we do not observe broad depletion of virus-susceptible cell populations, we detect increased expression of cell death–associated genes, which suggests that the host antiviral response is cytotoxic and may contribute to disease pathogenesis. Further investigation is warranted to determine whether similar responses occur in other SARS-CoV-2 target tissues both in vitro and in vivo.

## Materials and methods

### Air–liquid interface culture of HBECs

HBECs, from Lonza, were cultured in suspension in PneumaCult-Ex Plus Medium according to manufacturer’s instructions (STEMCELL Technologies, Cambridge, Massachusetts, USA). To generate ALI cultures, HBECs were plated on collagen-coated transwell inserts with a 0.4-micron pore size (Costar, Corning, Tewksbury, Massachusetts, USA) at 5 × 10^4^ cells/ml per filter and inserted into 24-well culture plates. Cells were maintained for the first 3 days in PneumaCult-Ex Plus Medium, then changed to PneumaCult-ALI Medium (STEMCELL Technologies) containing the ROCK inhibitor Y-27632 for 4 days. Fresh medium, 100 μl in the apical chamber and 500 μl in the basal chamber, was provided every day. On day 7, medium at the apical chambers were removed, while basal chambers were maintained with 500 μl of PneumaCult-ALI Medium. HBECs were maintained at an ALI for 28 days, allowing them to differentiate. Medium in the basal chamber was changed every 2 to 3 days (500 μl).

### Viral infection

SARS-CoV-2 isolate USA-WA1/2020 was obtained from BEI reagent repository. All infection experiments were performed in a Biosafety Level 3 facility, licensed by the State of Connecticut and Yale University. Immediately prior to infection, the apical side of the HBEC ALI culture was gently rinsed 3 times with 200 μl of phosphate buffered saline without divalent cations (PBS−/−). Moreover, 10^4^ PFUs of SARS-CoV-2 in 100 μl total volume of PBS was added to the apical compartment. Approximately 10^6^ cells were present at the time of infection in each sample yielding an MOI of approximately 0.01. Cells were incubated at 37°C and 5% CO_2_ for 1 hour. Unbound virus was removed, and cells were cultured with an ALI for up to 3 days. Infections were staggered by 1 day, and all 4 samples were processed simultaneously for scRNA-seq, as described below.

### Sample preparation for single-cell RNA sequencing

Inoculated HBECs were washed with 1X PBS−/− and trypsinized with TrypLE Express Enzyme (Thermo Fisher Scientific, Waltham, Massachusetts, USA) to generate single-cell suspensions. A total of 100 μl of TrypLE was added on the apical chamber, was incubated for 10 minutes at 37°C in a CO_2_ incubator, and was gently pipetted up and down to dissociate cells. Harvested cells were transferred in a sterile 1.5-ml tube and neutralized with DMEM containing 10% FBS. An additional 100 μl of TrypLE was placed on the apical chamber repeating the same procedure as above for a total of 30 minutes to maximize collection of cells. Cells were centrifuged at 300 × *g* for 3 minutes and resuspended in 100 μl DMEM with 10% FBS. Cell count and viability was determined using trypan blue dye exclusion on a Countess II (Thermo Fisher Scientific). The targeted cell input was 10,000 cells per condition, and based on calculations by 10x Genomics, we estimate our doublet rate is approximately 7.6%, given the cell numbers collected for each sample. The Chromium Next Gel Bead-In Emulsion (GEM) Single Cell 3’ Gel beads v3.1 kit (10x Genomics, Pleasanton, California, USA) was used to create GEMs following the manufacturer’s instructions. All samples and reagents were prepared and loaded into the chip and ran in the Chromium Controller for GEM generation and barcoding. GEMs generated were used for cDNA synthesis and library preparation using the Chromium Single Cell 3’ Library Kit v3.1 (10x Genomics) following the manufacturer’s instruction. Generated libraries were sequenced on NovaSeq 6000 system using HiSeq 100 base pair reads and dual indexing. Cells were sequenced to an average depth of 31,383 reads per cell. The human genome, Ensembl GRCh38.98.gtf, and the SARS-CoV-2 genome, NCBI Genome database accession MT020880.1, were combined and used for alignment. We ran the standard 10x Genomics cellranger pipeline with a combined human and SARS-CoV-2 genome to obtain count matrices for each of the 4 growth conditions. Per condition, there were an average of between 10,000 and 15,000 counts per cell or an average of 2,400 to 3,600 unique genes detected per condition. Library preparations and sequencing were performed by the Yale Center for Genome Analysis.

### Quantitative RT-PCR of SARS-CoV-2

Viral RNA from SARS-CoV-2–infected HBEC cell lysates was extracted using TRIzol (Life Technologies, Carlsbad, California, USA) and purified using Direct-zol RNA MiniPrep Plus according to the manufacturer’s instructions (Zymo Research, Irvine, California, USA). A 2-step cDNA synthesis with 5 μl RNA, random hexamer, and ImProm-II Reverse Transcriptase (Promega, Madison, Wisconsin, USA) was performed. The quantitative polymerase chain reaction (qPCR) analysis was performed in duplicate for each of the samples and standard curves generated using SARS-CoV-2 nucleocapsid (N1) specific oligonucleotides from Integrated DNA Technologies (Coralville, Iowa, USA): Probe: 5′ 6FAM-ACCCCGCATTACGTTTGGTGGACC-BHQ1 3′; Forward primer: 5′ GACCCCAAAATCAGCGAAAT-3′; Reverse primer: 5′ TCTGGTTACTGCCAGTTGAATCTG 3′. The limit of detection was 10 SARS-CoV-2 genome copies/μl. The virus copy numbers were quantified using a control plasmid which contains the complete nucleocapsid gene from SARS-CoV-2.

### Validation of polyadenylated SARS-CoV-2 transcripts

Huh7.5 cells grown in DMEM containing 10% FBS were infected with 10^4^ PFU of SARS-CoV-2, and cell lysates were harvested at 0, 1, 2, and 3 dpi. Using 0.3 μg total RNA extracted from mock or SARS- CoV-2-infected Huh7.5 cells at different time points, reverse transcription was performed with oligo-d(T)20 (Thermo Fisher Scientific) and MarathonRT, a highly processive group II intron-encoded RT. MarathonRT purification and RT reactions were performed as previously described [58]. PCR (NEBNext Ultra II Q5 R Master Mix, NEB, Ipswich, Massachusetts, USA) was performed with a gene-specific forward primer designed 700-nt upstream of the apparent boundary between the SARS-CoV-2 genome body and the putative poly-A tail. Oligo-d(T)20 was used as a reverse primer. Touchdown PCR cycling was used to enhance specificity of the PCR reaction. RT-PCR products were resolved on a 1.3% agarose gel with ladder (100 bp DNA Ladder, 1 kb Plus DNA Ladder, Invitrogen, Carlsbad, California, USA). Forward PCR oligonucleotides used in this experiment are below, which includes 2 positive controls. Primer Name Position on Genome 5′-3′ Sequence:

F Val 1 7700 GAGAGACTTGTCACTACAGTTTAAA

F Val 2 26650 AATTTGCCTATGCCAACAGGA

F Val (+)ve 1 28600 AGATCTCAGTCCAAGATGGTA

F Val (+)ve 2 29000 GGTAAAGGCCAACAACAACAA

### Electron microscopy

The cells were fixed using 2.5% glutaraldehyde in 0.1 M phosphate buffer, osmicated in 1% osmium tetroxide, and dehydrated in increasing ethanol concentrations. During dehydration, 1% uranyl acetate was added to the 70% ethanol to enhance ultrastructural membrane contrast. After dehydration, the cells were embedded in Durcupan. Moreover, 70-nm ultrathin sections were cut on a Leica ultramicrotome, collected on Formvar coated single-slot grids, and analyzed with a Tecnai 12 Biotwin electron microscope (FEI, Hillsboro, Oregon, USA).

### Immunofluorescence assay

HBECs grown in transwell filters as described above were inoculated with 10^4^ PFU of ic-SARS-CoV-2-mNG [59] or a mock control. HBECs were fixed with 4% PFA for 30 minutes at RT, followed by permeabilization with 0.2% Triton X100 in 1X PBS for 10 minutes at RT. Cells were blocked with 10% normal goat serum in 1X PBS (blocking buffer) for 1 hour at RT. Primary antibodies for Ac-tubulin (Abcam, Cambridge, Massachusetts, USA) and Forkhead Box J1 (FOXJ1) (Sigma Aldrich, St. Louis, Missouri, USA) were diluted in blocking buffer at 1:500 and were incubated overnight at 4°C. Goat anti-mouse Alexa Fluor 594 (BioLegend, San Diego, California, USA) and goat anti-rabbit APC (Invitrogen) were diluted in the blocking buffer at 1:200 and were applied for 2 hours at RT and further stained with Hoechst 33342 (Life Technologies) for 30 minutes at RT. The transwell filters were then cut and placed in a glass slide and mounted with a Prolong Diamond Antifade Mountant (Life Technologies). Representative photos were taken using a Leica LSR microscope. Scale bars correspond to 25 μm.

### Cytokine measurement by multiplex immunoassay

Levels of IL-6, IL-1A, IL-1B, IL-1RN, and CXCL9 in the basolateral supernatants of mock and infected HBECs from 3 independent experiments were all performed by EVE technologies (Calgary, Alberta, Canada) using the multiplex immunoassay analyzed with a BioPlex 200. All statistical analysis was performed using Prism GraphPad version 8. All were statistically analyzed using nonparametric Kruskal–Wallis test is indicated with a bar, and the *p*-value is represented by a symbol (*p < 0.05, **p < 0.01, ***p < 0.001).

### scRNA-seq data analysis

#### Cell type annotation

We used the standard scRNA-seq analysis pipeline for clustering [60]. Briefly, to account for transcript dropout inherent to scRNA-seq, we removed genes that were expressed in fewer than 3 cells and removed cells that expressed fewer than 200 genes. Next, we filter out cells with more than 10% of mitochondrial genes. We did not find a correlation between viral copy number and mitochondrial expression. The resulting raw unique molecular identifier (UMI) counts in each cell were normalized to their library size. Then, normalized counts were square-root transformed, which is similar to a log transform but does not require addition of a pseudo count. Data preprocessing was performed in Python (version 3.7.4) using Scanpy (version 1.4.6) [61,62]. We visually observed batch effects between conditions in 2D cellular embeddings. To remove these batch effects for clustering, cell type annotation, and visualization, we used an approximate batch-balanced kNN graph for manifold learning (BB-kNN batch-effect correction) using Scanpy’s fast approximation implementation [61,62]. BB-kNN assumes that at least some cell types are shared across batches and that differences between batches for a same cell type are lower than differences between cells of different types within a batch. For each cell, the 3 nearest neighboring cells in each condition were identified by Euclidean distance in 100-dimensional principal component analysis (PCA) space. This kNN graph was used as the basis for downstream analysis. To visualize the scRNA-seq data, we implemented various nonlinear dimension reduction methods and used the BB-kNN batch-corrected connectivity matrix as input for UMAP [63] and Potential of Heat-diffusion for Affinity-based Trajectory Embedding (PHATE) [64]. UMAP projections were generated using a minimum distance of 0.5. PHATE projections were generated with a gamma parameter of 1. For cell clustering, we used the Louvain community detection method [65] with the BB-kNN graph. We used high-resolution community detection and merged clusters based on expression of bronchial epithelium cell-type markers in order to isolate some rare cell types, e.g., tuft cells [66,67]. To annotate the different cell types present in HBECs, we analyzed expressions of a range of marker genes that were reported [67–77] and in a molecular cell atlas from Travaglini colleagues [66]. We focused on 8 cell types: (i) basal cells (KRT5, DAPL1, and TP63); (ii) ciliated cells (FOXJ1, CCDC153, CCDC113, MLF1, and LZTFL1); (iii) club cells (SCGB1A1, KRT15, CYP2F2, LYPD2, and CBR2); (iv) BC/club (KRT4 and KRT13); (v) neuroendocrine cells (CHG1 and ASCL1); (vi) Tuft cells (POU2F3, AVIL, GNAT3, and TRPM5); (vi) ionocytes (FOXI1, CFTR, and ASCL3); and (viii) goblet cells (MUC5AC, MUC5B, GP2, and SPDEF).

#### Infection threshold and metric for transcriptomic similarity to infected cells

Counting a viral transcript in a cell does not mean the cell is infected, as this count can come from a virus attached to the surface of the cell, ambient virus in the suspension, or from read misalignment. Given the reported shared 3′ poly(A) tail in coronavirus transcripts [78], we were unsure whether we could correctly capture the different ORFs using the 10x Genomics 3′ gene expression library. Therefore, we aligned the viral reads to a genome-wide single “exon,” i.e., a count is given for a read mapped to SARS-CoV-2 ORFs and intergenic regions. These counts were used to infer individual cells’ infectious state. To filter out cells with viral genome transcript counts that may result from viral cell surface attachment, ambient virus in the droplet suspension, or read misalignment, we considered infected cells to have 10 viral transcripts counts. This threshold of 10 viral transcripts to define an infected cell was determined empirically as it represents an inflection point (S1A Fig). While the mock condition is not expected to have viral counts, we did observe a small number of reads that could be attributed to misalignment or transcript leakage. We observed only 5 mock cells with full SARS-CoV-2 viral genome transcript counts 10 transcripts. These criteria allowed us to find 144 infected cells at 1 dpi, 1,428 cells at 2 dpi, and 3,173 cells at 3 dpi. To quantify the extent to which an individual cell is transcriptionally similar to an infected cell, we used a previously developed graph signal processing approach called Manifold Enhancement of Latent Dimensions (MELD) [79]. We encoded a raw experimental score for each cell in the dataset such that −1 represents a bystander or uninfected cell, and +1 represents an infected cell. Using the kernel from the BB-kNN graph (described above), these raw scores were smoothed in the graph domain, yielding a metric for transcriptomic similarity to infected cells per cell that represents the extent to which an individual cell is transcriptionally similar to infected cells. For example, if an infected cell is more transcriptionally similar to bystander cells, it will have a low value of the metric, closer to −1. Cells in a cluster of transcriptionally similar cells that are infected will have values closer to +1, indicating similar transcriptomic signatures to infected cells. For summary statistics, this score was stratified by cell type and condition.

#### Viral genome read coverage analysis

To visualize the viral read coverage along the viral genome, we used the 10x Genomics cellranger barcoded binary alignment map (BAM) files for every sample. We filtered the BAM files to only retain reads mapping to the viral genome using the bedtools intersect tool [80]. We converted the BAM files into sequence alignment map (SAM) files in order to filter out cells that were removed in our single-cell data preprocessing pipeline. The sequencing depth for each base position was calculated using samtools count. To characterize read distribution along the viral genome, we counted transcripts of 10 different ORFs: ORF1ab, Surface glycoprotein (S), ORF3a, Envelope protein (E), Membrane glycoprotein (M), ORF6, ORF7a, ORF8, Nucleocapsid phosphoprotein (N), and ORF10.

#### Differential expression analysis

To find DEGs across conditions, we used a combination of 3 metrics: the Wasserstein or Earth Mover’s Distance, an adjusted *p*-value from a 2-sided Mann–Whitney U test with continuity and Benjamini–Hochberg correction, and the binary logarithm of fold change between mean counts. Significance was set to *p*_*adjusted*_ 0.01. The Earth Mover’s Distance, or 1-dimensional Wasserstein distance, can be defined as the minimal cost to transform distribution to another and was previously used to assess gene expression that significantly differs between conditions [81,82]. We performed several binary comparisons for each time point and for pooling 1, 2, and 3 dpi: infected versus bystander, infected versus mock cells, and bystander versus mock cells. The 30 most DEGs (up- or down-regulated, ranked by Wasserstein distance) in each condition, cell type, and analysis were represented in heatmaps. To identify up-regulated and down-regulated cellular pathways across conditions in infected and bystander ciliated cells, we analyzed the top and bottom 200 DEGs using PANTHER-GO [83] statistical overrepresentation tests utilizing the default Human PANTHER-GO reference list as a background. Statistical significance was assessed by Fisher exact test, and correction was assessed by calculating the false discovery rate.

## Supporting information

S1 Fig Detection of SARS-CoV-2 viral transcript and genome in different cell types. **(A)** Margin for which SARS-CoV-2 preferentially infects ciliated cells for various thresholds. The margin of the difference in SARS-CoV-2 tropism for various cell types across each time point was calculated by taking the percent of infected ciliated cells for each threshold minus the percent infected of the highest infected, non-ciliated cell type. Line style shows margin of ciliated cell tropism for each time point, and colored points show the highest infected non-ciliated cell type for each infection threshold and condition. **(B)** For all thresholds based on read counts aligned to the SARS-CoV-2 genome, the percentage of thresholds for which ciliated cells are the highest infected cell type across each time point. **(C)** Histogram of the average raw counts of viral transcripts per cell type across conditions in a given time point. **(D)** Infection score inferred from MELD showing prototypicality of infection per cell, stratified by condition (color). **(E)** Heatmap, range-scaled for each row (cell type), where the color represents expression (normalized and square-root transformed counts) of viral ORFs in each cell type across 3 conditions: 1, 2, and 3 dpi. The individual numerical value per condition for A–E is listed in S1 Data. CC, ciliated cell type; dpi, days post-infection; non-CC, a cell type that is not a ciliated cell; MELD, Manifold Enhancement of Latent Dimensions; ORF, open reading frame; SARS-CoV-2, Severe Acute Respiratory Syndrome Coronavirus 2.
(TIF)

S2 Fig Innate immune response in different cell types in SARS-CoV-2 infection. **(A–D)** Heatmap showing expression of key innate immune and inflammatory genes in neuroendocrine cells **(A)**, ionocytes **(B)**, tuft **(C)**, and goblet **(D)** in infected, bystander, and uninfected cells at different time points. The color scale shows the average expression (represented as z-score) for each cell type and condition. The individual numerical value per condition for A–D is listed in S1 Data. SARS-CoV-2, Severe Acute Respiratory Syndrome Coronavirus 2.
(TIF)

S3 Fig DEGs across different cell types. **(A)** Heatmaps showing the average expression (represented as z-score) of the union of the top 30 most differentially up-regulated and top 30 most differentially down-regulated genes between infected and bystander cells in each condition. **(B)** Schematic of the differential expression analysis, comparing infected and mock ciliated cells at 1, 2, and 3 dpi. **(C)** Schematic of the differential expression analysis comparing mock and bystander ciliated cells at 1, 2, and 3 dpi. (B, C) The volcano plots annotate the top 10 up-regulated and down-regulated genes between mock and infected ciliated cells, as ranked by EMD, after pooling cells from 1, 2, and 3 dpi. The individual numerical value per condition for A–C is listed in S1 Data. DEG, differentially expressed gene; dpi, days post-infection; EMD, Earth Mover’s Distance.
(TIF)

S4 Fig DEG analysis in ciliated cells at different time points. Infected versus bystander differential gene expression analysis in ciliated cells, separated for each time point: 1 dpi **(A)**, 2 dpi **(B)**, and 3 dpi **(C)**. **(D)** Overlap in the number of genes that are significantly differentially expressed in ciliated cells across time points. Significance is defined as *P* corrected (Benjamini–Hochberg) < = 0.01. The individual numerical value per condition for A–C is listed in S1 Data. The raw data for generating A–D are listed in S2 Data. DEG, differentially expressed gene; dpi, days post-infection.
(TIF)

S1 Data The individual numerical values for the following figure panels: Figs 1B, 1C, 1D, 1E, 1F, 1G, 2A, 2B, 2C, 3A, 3B, 3C, 3D, 4A, 4B, 4C, 4D, 4E, 4F, 4G, 4H, 4I, 5A, 5B, 5C, 5D, 6B, 6C and 6D and S1A, S1B, S1C, S1D, S1E, S2A, S2B, S2C, S2D, S3A, S3B, S3C, S4A, S4B, S4C and S4D Figs. (XLSX)

S2 Data The raw data used to generate S4A, S4B, S4C and S4D Figs. (XLSX)

S3 Data The raw data used to generate Fig 6B, 6C and 6D. (XLSX)

S1 Raw Images The raw images for Fig 2D. (TIF)

S2 Raw Images The raw images for Fig 3E. (TIF)

S3 Raw Images The raw images for Fig 3F. (TIF)

## Abbreviations

Ac-tubulin: acetylated tubulin
ACE2: angiotensin converting enzyme II
ALI, air: liquid interface
BAM: binary alignment map
BB-kNN: batch-balanced kNN
CoV: Coronavirus
COVID-19: Coronavirus Disease 2019
DEG: differentially expressed gene
dpi: days post-infection
FDA: Food and Drug Administration
FOXJ1: Forkhead Box J1
GEM: Gel Bead-In Emulsion
HBEC: human bronchial epithelial cell
IFN: interferon
IL: interleukin
ISG: interferon-stimulated gene
MELD: Manifold Enhancement of Latent Dimensions
MERS-CoV: Middle East Respiratory Syndrome Coronavirus
MOI: multiplicity of infection
nsp8: nonstructural protein 8
ORF: open reading frame
PCA: principal component analysis
PFU: plaque forming unit
PHATE: Potential of Heat-diffusion for Affinity-based Trajectory Embedding
qPCR: quantitative polymerase chain reaction
qRT-PCR: quantitative real-time polymerase chain reaction
RNA-seq: RNA sequencing
RT-PCR: reverse transcription PCR
SAM: sequence alignment map
SARS: Severe Acute Respiratory Syndrome
SARS-CoV-2: Severe Acute Respiratory Syndrome Coronavirus 2
sc: single-cell
sgRNA: subgenomic RNA
TMPRSS2: transmembrane protease serine 2
TNFα: tumor necrosis factor alpha
TRS: transcription regulatory sequence
UMAP: Uniform Manifold Approximation and Projection
UMI: unique molecular identifier
