# Supplementary figures and images for: Single-cell longitudinal analysis of SARS-CoV-2 infection in human airway epithelium identifies target cells, alterations in gene expression, and cell state changes
Source: PLoS Biol. 2021 Mar 17;19(3):e3001143. doi: 10.1371/journal.pbio.3001143 (PMC8007021; doi:10.1371/journal.pbio.3001143)

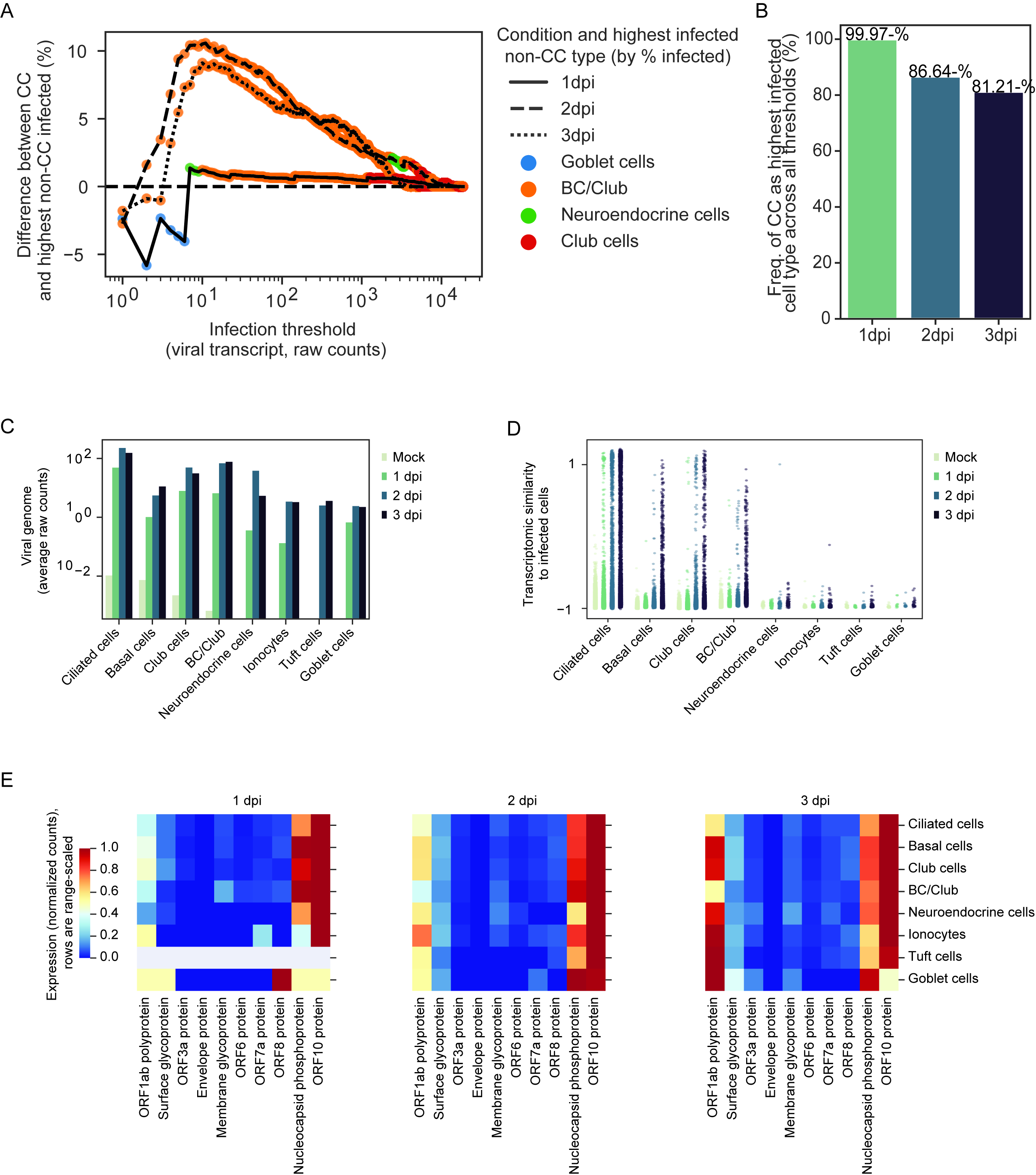

Supplement: S1 Fig — (A) Margin for which SARS-CoV-2 preferentially infects ciliated cells for various thresholds. The margin of the difference in SARS-CoV-2 tropism for various cell types across each time point was calculated by taking the percent of infected ciliated cells for each threshold minus the percent infected of the highest infected, non-ciliated cell type. Line style shows margin of ciliated cell tropism for each time point, and colored points show the highest infected non-ciliated cell type for each infection threshold and condition. (B) For all thresholds based on read counts aligned to the SARS-CoV-2 genome, the percentage of thresholds for which ciliated cells are the highest infected cell type across each time point. (C) Histogram of the average raw counts of viral transcripts per cell type across conditions in a given time point. (D) Infection score inferred from MELD showing prototypicality of infection per cell, stratified by condition (color). (E) Heatmap, range-scaled for each row (cell type), where the color represents expression (normalized and square-root transformed counts) of viral ORFs in each cell type across 3 conditions: 1, 2, and 3 dpi. The individual numerical value per condition for A–E is listed in S1 Data. CC, ciliated cell type; dpi, days post-infection; non-CC, a cell type that is not a ciliated cell; MELD, Manifold Enhancement of Latent Dimensions; ORF, open reading frame; SARS-CoV-2, Severe Acute Respiratory Syndrome Coronavirus 2. (TIF) [file pbio.3001143.s001.tif]

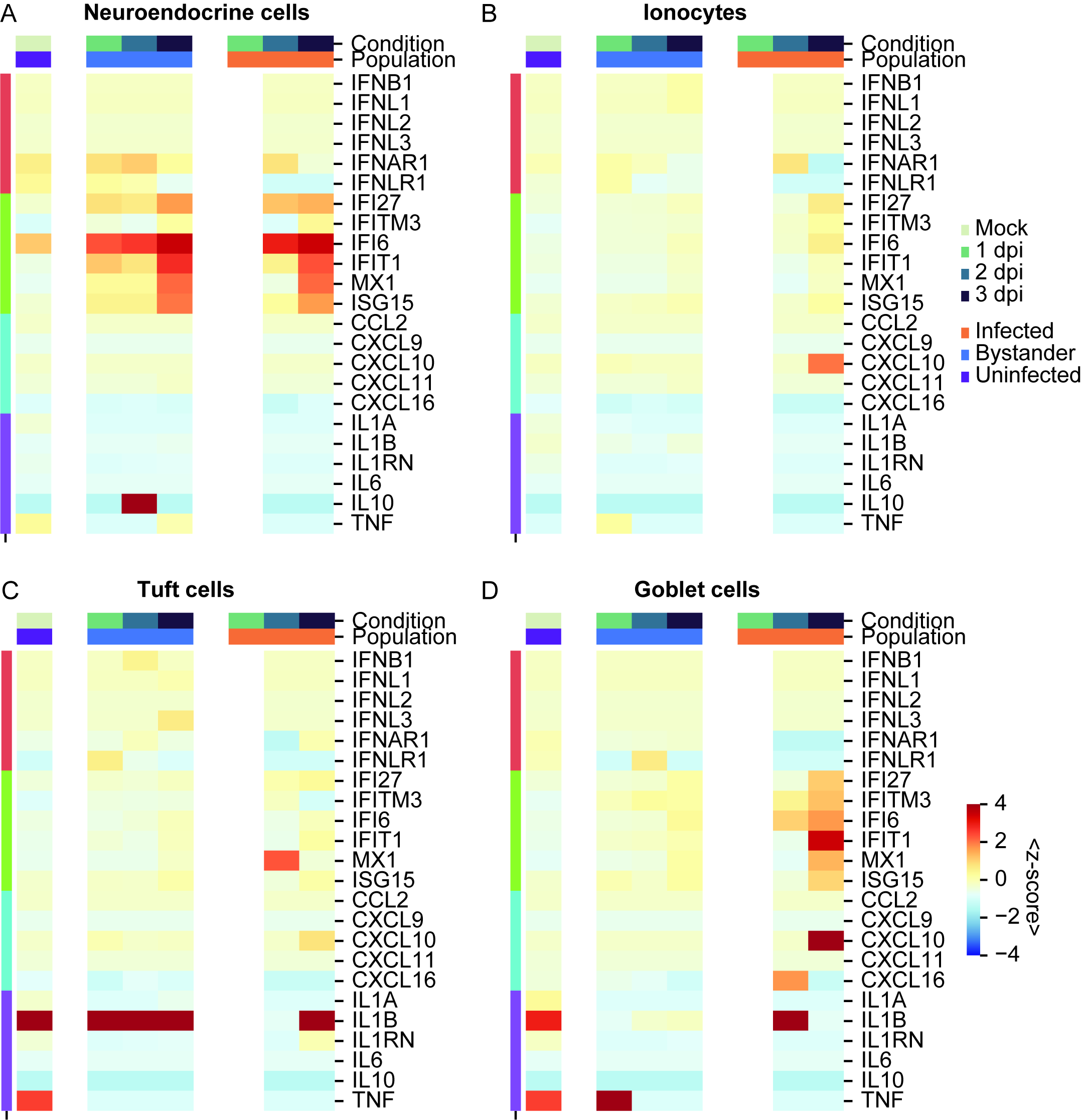

Supplement: S2 Fig — (A–D) Heatmap showing expression of key innate immune and inflammatory genes in neuroendocrine cells (A), ionocytes (B), tuft (C), and goblet (D) in infected, bystander, and uninfected cells at different time points. The color scale shows the average expression (represented as z-score) for each cell type and condition. The individual numerical value per condition for A–D is listed in S1 Data. SARS-CoV-2, Severe Acute Respiratory Syndrome Coronavirus 2. (TIF) [file pbio.3001143.s002.tif]

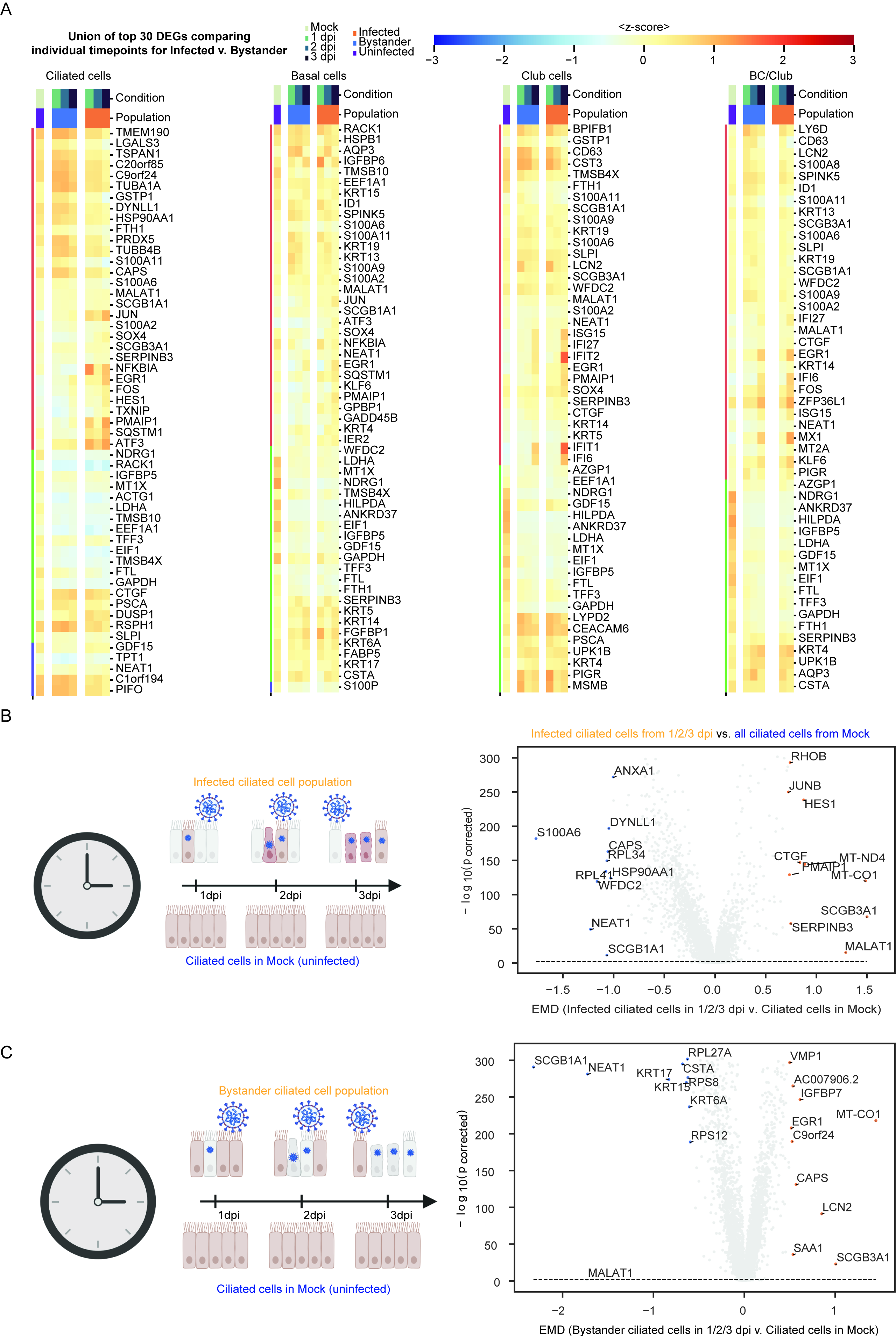

Supplement: S3 Fig — (A) Heatmaps showing the average expression (represented as z-score) of the union of the top 30 most differentially up-regulated and top 30 most differentially down-regulated genes between infected and bystander cells in each condition. (B) Schematic of the differential expression analysis, comparing infected and mock ciliated cells at 1, 2, and 3 dpi. (C) Schematic of the differential expression analysis comparing mock and bystander ciliated cells at 1, 2, and 3 dpi. (B, C) The volcano plots annotate the top 10 up-regulated and down-regulated genes between mock and infected ciliated cells, as ranked by EMD, after pooling cells from 1, 2, and 3 dpi. The individual numerical value per condition for A–C is listed in S1 Data. DEG, differentially expressed gene; dpi, days post-infection; EMD, Earth Mover’s Distance. (TIF) [file pbio.3001143.s003.tif]

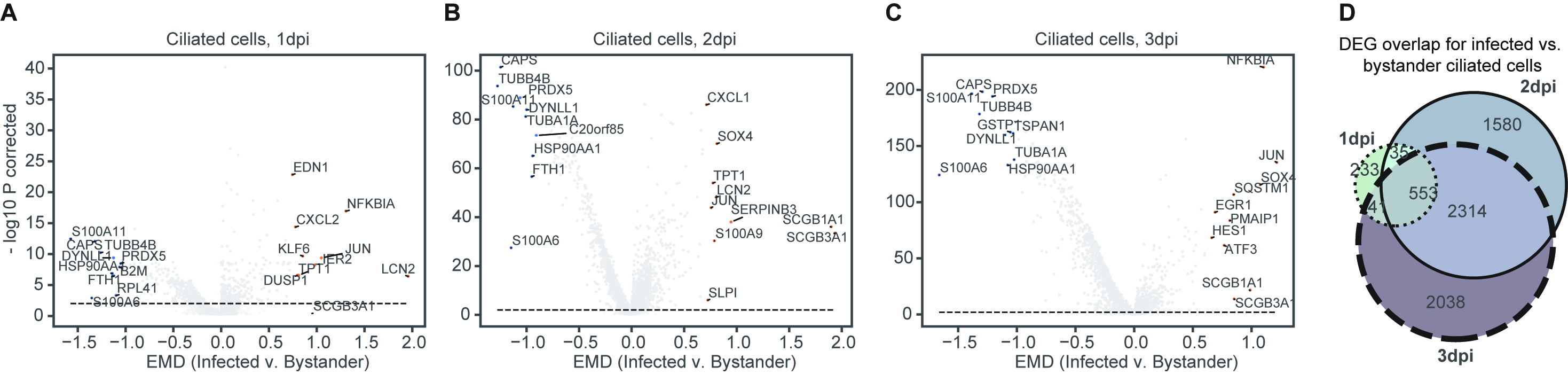

Supplement: S4 Fig — Infected versus bystander differential gene expression analysis in ciliated cells, separated for each time point: 1 dpi (A), 2 dpi (B), and 3 dpi (C). (D) Overlap in the number of genes that are significantly differentially expressed in ciliated cells across time points. Significance is defined as P corrected (Benjamini–Hochberg) < = 0.01. The individual numerical value per condition for A–C is listed in S1 Data. The raw data for generating A–D are listed in S2 Data. DEG, differentially expressed gene; dpi, days post-infection. (TIF) [file pbio.3001143.s004.tif]

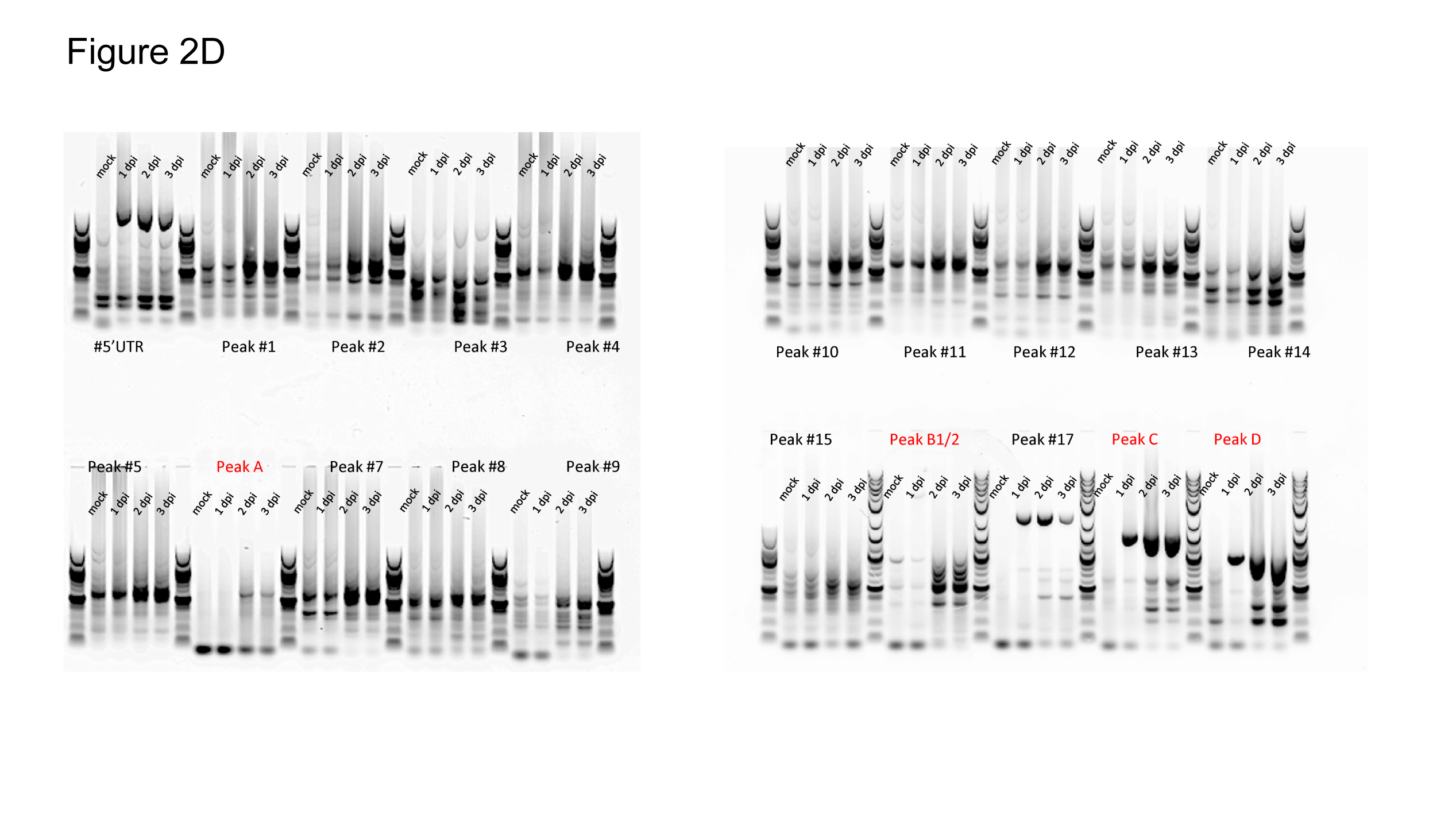

Supplement: S1 Raw Images — (TIF) [file pbio.3001143.s008.tif]

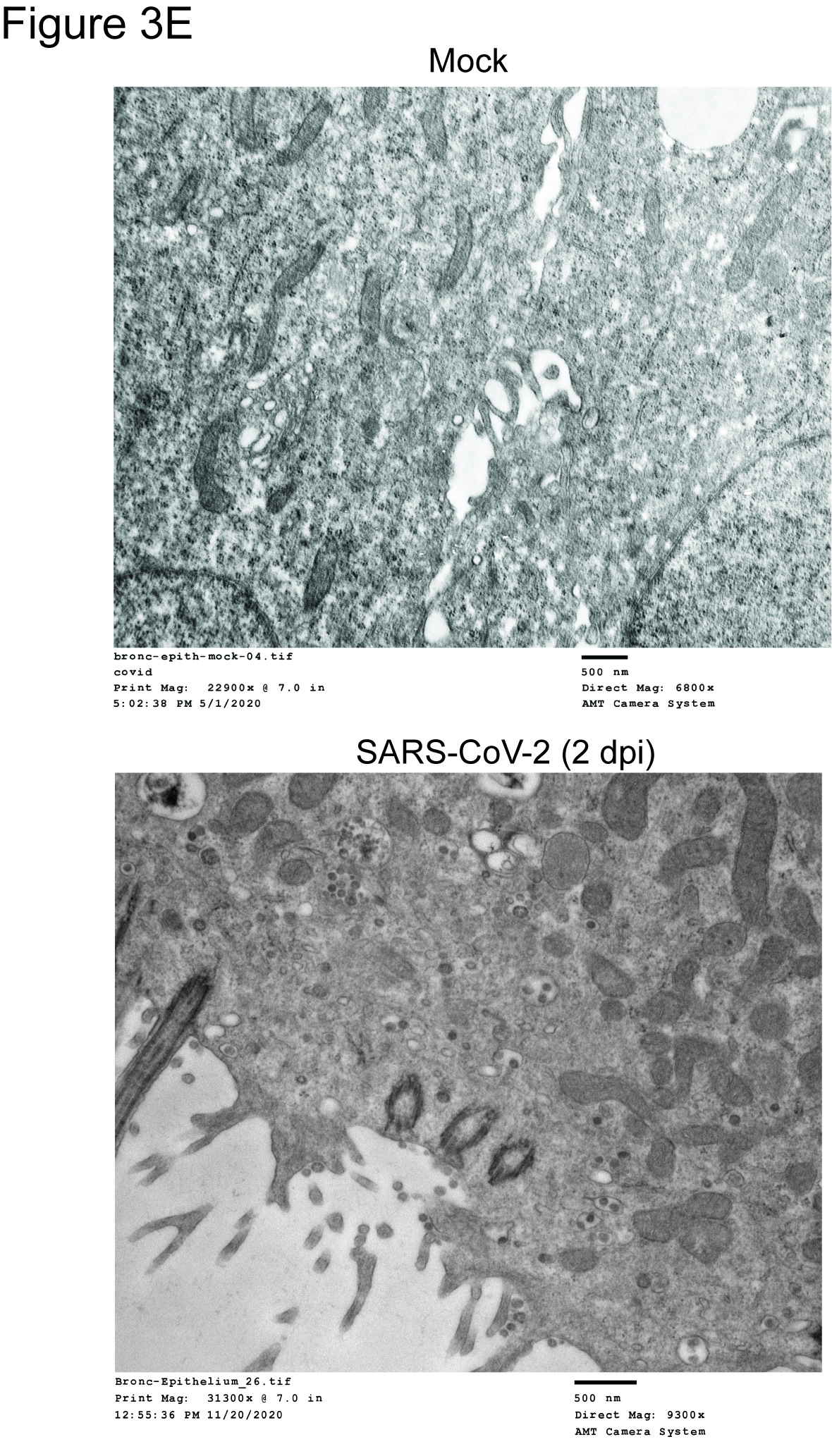

Supplement: S2 Raw Images — (TIF) [file pbio.3001143.s009.tif]

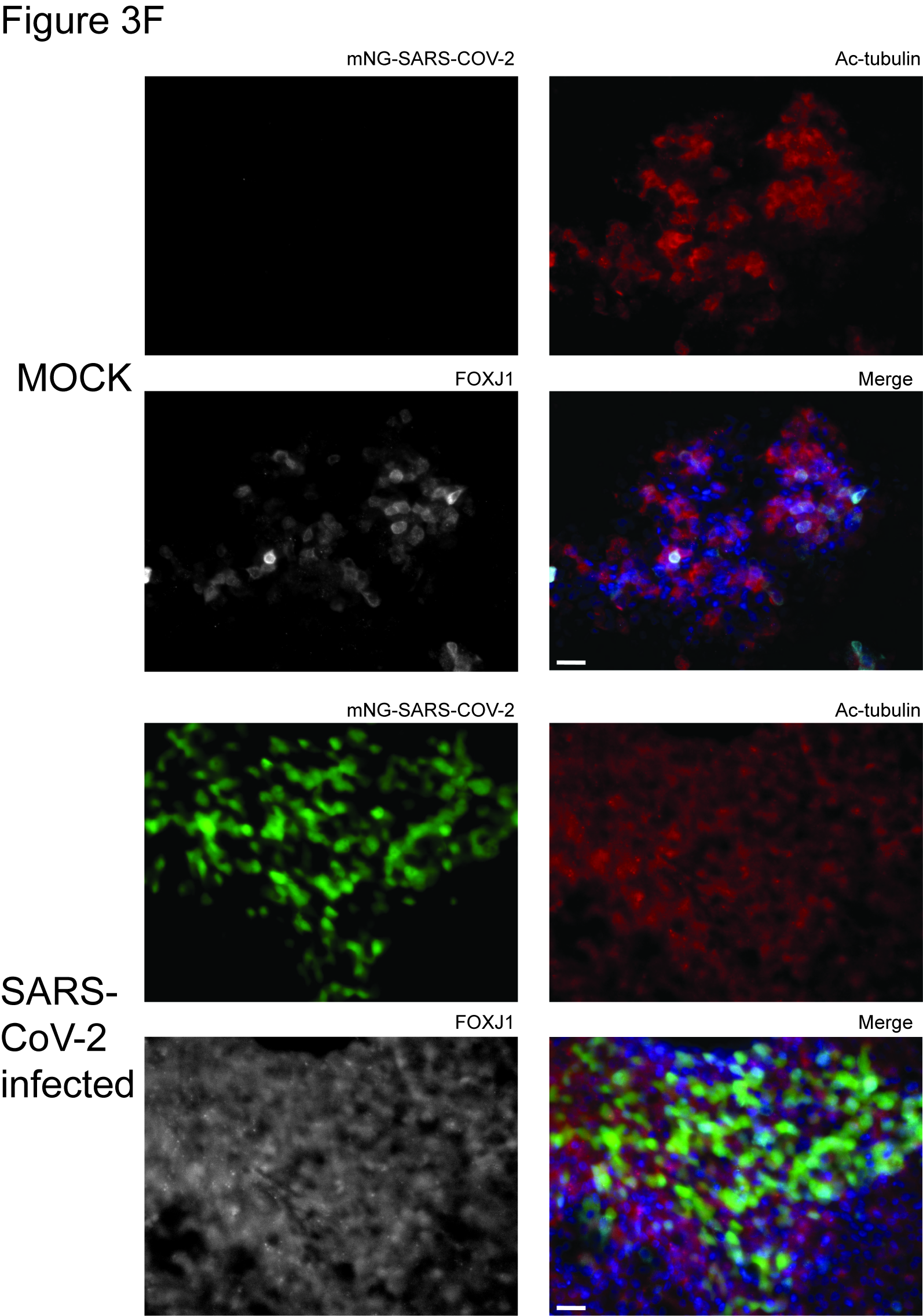

Supplement: S3 Raw Images — (TIF) [file pbio.3001143.s010.tif]
